# Evolution of Precipitated Phases during Creep of G115/Sanicro25 Dissimilar Steel Welded Joints

**DOI:** 10.3390/ma14175018

**Published:** 2021-09-02

**Authors:** Maohong Yang, Zheng Zhang, Linping Li

**Affiliations:** 1 School of Material Science and Engineering, Beihang University, 37 Xueyuan Road, Beijing 100191, China; y970094835@163.com; 2 Shenhua Guohua (Beijing) Electric Power Research Institute Co., Ltd., No.75 Jian Guo Road, Chaoyang District, Beijing 100025, China; linping.li@chnenergy.com.cn

**Keywords:** dissimilar steel welded joint, creep, laves phase, cavity

## Abstract

This paper studies the evolution of the microstructure and microhardness in the G115 side of the G115/Sanicro25 dissimilar steel welded joint during the creep process. The joints were subjected to creep tests at 675 °C, 140 MPa, 120 MPa and 100 MPa. A scanning electron microscope equipped with an electron backscattering diffraction camera was used to observe the microstructure of the cross-section. The fracture position of the joint and the relationship between the cavity and the second phase were analyzed. The microstructure morphology of the fracture, the base metal and the thread end was compared and the composition and size of the Laves phase were statistically analyzed. The results show that the fracture locations are all located in the fine-grain heat-affected zone (FGHAZ) zone, and the microstructure near the fracture is tempered martensite. There are two kinds of cavity in the fracture section. Small cavities sprout adjacent to the Laves phase; while large cavities occupy the entire prior austenite grain, there are more precipitated phases around the cavities. The Laves phase nucleates at the boundary of the M_23_C_6_ carbide and gradually grows up by merging the M_23_C_6_ carbide. Creep accelerates the coarsening rate of the Laves phase; aging increases the content of W element in the Laves phase.

## 1. Introduction

High-parameter ultra-supercritical thermal power plants are currently the first choice for clean and efficient power generation. Studies have shown that the thermal efficiency of the unit can be increased by about 0.25% for every 10 °C increase in the main steam temperature [1]. However, the improvement of steam parameters, such as temperature and pressure, is largely restricted by the performance of high-temperature heat-resistant materials. As a candidate material for the 650 °C parameter unit, G115 steel has made certain improvements to the composition of MARBN steel on the basis of Abe et al. [2] and combined with the existing research results of traditional 9%–12% Cr steel. It has excellent microstructure stability performance, high temperature creep performance and steam oxidation resistance in the range 630–650 °C. Its creep rupture strength at 650 °C is 1.5 times that of P92 steel, and its high temperature steam oxidation resistance and weldability are equivalent to P92 steel [3]. At present, the creep and oxidation resistance properties of G115 have been studied. However, there are few reports on its join to austenitic steel and creep properties. This severely restricts the development of high-parameter ultra-supercritical units at 630–650 °C. Therefore, there is an urgent need for a more reasonable and mature welding technology and corresponding high-temperature performance corresponding to the new heat-resistant steel.

In a few cases, the Laves phase precipitated after heat treatment [4]. In most cases, the Laves phase precipitates during high temperature aging and creep [5,6]. The size of the observable Laves phase is about 200 nm [6]. This phenomenon has been verified in the literature [7], where large Laves phase particles suddenly appeared after 2000 h. There are currently two different views on the nucleation method of the Laves phase. Xu et al. [8] reported two nucleation methods of Laves phase in 10.46 Cr steel. One is that the Laves phase nucleate near the M_23_C_6_ carbide, and then gradually swallow the M_23_C_6_ carbide. The other is that the Laves phase nucleates alone on the prior-austenite grain boundary or the martensite lath boundary. Eggeler et al. [9,10] held a different view. They believed that the Laves phase nucleated near M_23_C_6_ but grew along the micrograin boundary. The W element in martensitic heat-resistant steel is necessary to stabilize the microstructure [11]. The Laves phase is easily formed and grown in the delta ferrite matrix compared with lath martensite due to the different solubility limit of tungsten [12]. The contribution of Laves to the creep strength of heat-resistant steel depends on its size and distribution. The small-sized Laves phase is beneficial to the creep strength, while the large-sized Laves phase will damage the creep properties [13]. M_23_C_6_ carbides and Laves phases with a size greater than 0.5 μm are considered to have an adverse effect on the properties of the material [14]. It also provides nucleation sites of the crack and acts as a brittle phase. Literature [15,16,17], respectively, quantified the contribution of various strengthening methods in heat-resistant steel to its performance, and found that precipitation strengthening occupies approximately 15–30% of the strength. Earlier studies have shown that at 650 °C, the order of the mass fraction of stable phase in P92 steel is M_23_C_6_ > Laves phase > MX phase [15]; while at the same temperature, the order in G115 steel is Laves phase > M_23_C_6_ > Copper-rich phase > MX phase [18]. There are few studies on the influence of the mass fraction, element content and size of the stable phase on the mechanical properties in the welded joints of dissimilar steels, and the relationship between the creep cavities and the precipitated phases.

In this article, ErNiCrCoMo-1 welding wire was used to prepare G115/Sanicro25 dissimilar steel welded joints by Tungsten inert gas welding (TIG) welding. After the heat treatment of the welded joints, creep tests were carried out at different stress levels at 675 °C. Modern characterization methods were used to study the microstructure evolution of G115 side during creep. We focus on two aspects. The first is the evolution of the size and composition of the Laves phase under different creep times. The second is the evolution of the Laves phase in the fracture, the base metal and the thread end.

## 2. Materials and Methods

**Figure 1:**
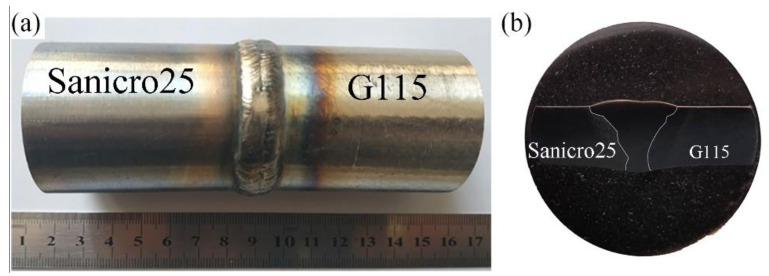
Welded joint: (**a**) Macro morphology of weld; (**b**) Weld cross section

The base metals (BM) on both sides of the dissimilar metal welds (DMWs) are G115 martensitic heat-resistant steel and Sanicro25 austenitic heat-resistant steel, and the nickel-based welding material (WM) named ERNiCrCoMo-1 is used to fill the weld. The chemical composition of BMs and WM is shown in Table 1. The dimeter of the BM is 44 mm, and the wall thickness is 7 mm. The weld is V-shaped, and the groove angle is 70°. The two BMs are preheated before welding. The preheating temperature is 150–200 °C. TIG is used for welding, the welding current is 90A, and the resulting joint is shown in Figure 1. Due to the difference in linear expansion coefficients of G115 and Sanicro25 steels, large residual stresses will be generated at the weld interface during welding. At the same time, the welded joint after welding has a solidified microstructure with loose and coarse grains, and is prone to metallurgical defects, such as pores and cracks. Therefore, post-weld heat treatment is performed to improve the microstructure and performance and remove residual stress. Post-weld heat treatment is performed on the joints, the heat treatment temperature is 780 °C, the heat preservation is 2 h, and it is cooled to room temperature in the air.

The axis of the creep specimen is perpendicular to the welding direction. The gauge length is 25 mm and the diameter is 5 mm. The weld is located in the middle of the gauge length section, and the axis of the specimen is located in the middle of the joint wall thickness. The creep test was carried out at 675 °C with 140 MPa, 120 MPa and 100 MPa stress respectively. The creep life and fracture position are shown in Table 2.

**Figure 2:**
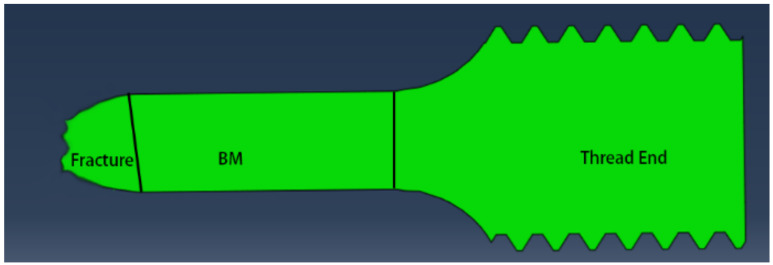
Schematic diagram of fracture section with fracture zone, BM zone and thread end zone.

For each creep specimen, the microstructure and microhardness of three parts on the side of G115 steel are mainly studied. The first part is near the fracture, the second part is the BM (6 mm away from the fracture), and the third part is the thread end (TE) of the creep specimen, as shown in Figure 2. The TE of the creep specimen has little stress and almost no strain accumulation during high temperature creep, which can be regarded as an aging state sample [6]. 

**Figure 3:**
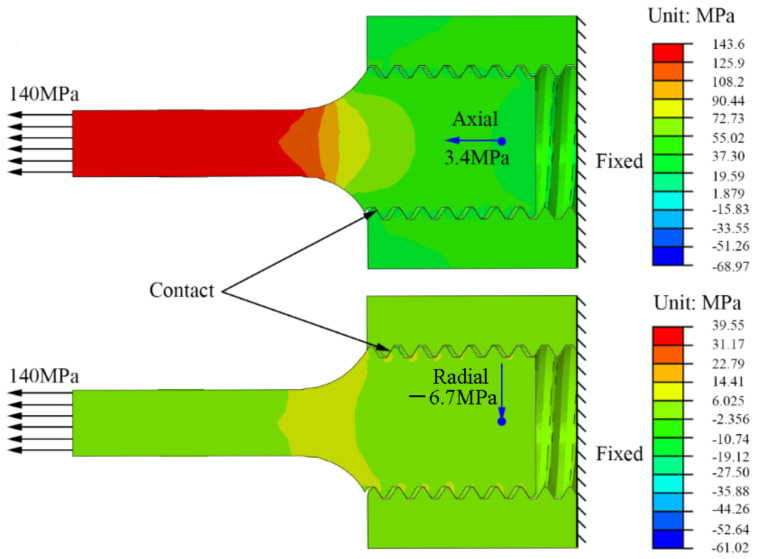
Axial and radial stress of G115 part of creep specimen.

At the same time, we also performed a finite element simulation on the stress distribution of the G115 part of the joint. The three-dimensional calculation model includes a part of the creep specimen and the fixture. The fixture is assumed to be a rigid body. The clamp and the sample are connected by contact, and the friction coefficient is 0.15. The freedom degree of displacement is fixed on one end face of the fixture, and the stress is applied on one end face of the specimen. In order to accurately calculate the stress distribution on the thread, the mesh of the thread teeth is refined, and the total number of mesh is 55,077, and the element type is C3D4. The test gauge length is 5 mm in diameter, the temperature is 675 °C, the elastic modulus of G115 steel is 165 GPa, the Poisson’s ratio is 0.3, and the applied stress is 140 MPa. The model is solved with commercial finite element software abaqus. The calculated results are shown in the Figure 3. It can be seen that the axial stress at the threaded end is 3.4 MPa, and the radial stress is −6.7 MPa. Therefore, it can be regarded as a sample in an aged state.

The fractured sample is cut along the axial direction. After polishing with water sandpaper, corroded with 5 g ferric chloride + 15 mL hydrochloric + 80 mL distilled water, and then the microstructure morphology was observed under ZEISS GeminiSEM 300 scanning electron microscope (SEM) (Carl Zeiss AG, Heidenheim, Germany). In the SEM analysis, the Laves phase particles in the sample are identified by the backscattering of electrons (BSE) (Carl Zeiss AG, Heidenheim, Germany) imaging mode. According to the differences of the average atomic number, the SEM-BSE image can clearly distinguish the M_23_C_6_ phase from the Laves phase. The latter is bright because of the large average atomic number. At the same time, the energy spectrometer (EDS) (Carl Zeiss AG, Heidenheim, Germany) is used to analyze the alloy composition of the precipitated particles. More than 10 particles for each precipitation phase are measured and the average value is taken (in order to avoid the mutual influence between adjacent particles, select separated particles in the field of view for measurement). The SEM-BSE image is binarized, and then the image processing software is used to quantify the Laves parameters. In order to ensure the statistical significance of the quantitative results, each sample is tested with two to three fields of view, the area fraction and number density are obtained, and the average particle diameter is calculated using the following formula:

(1) $$d=\sqrt{\frac{4f_{A}}{\pi \rho_{n}}}$$

where *f_A_* is the area fraction of the precipitated phase; *ρ_n_* is the number density of the precipitated phase.

The electron backscatter diffraction (EBSD) test was carried out on the FEI Sirion200 field emission SEM (FEI NanoPorts, Hillsboro, OR, USA). It was equipped with Oxford EBSD system, the EBSD analysis software was HKL channel 5, and the test voltage was 20 kV. The EBSD scan step size is 0.02 μm. The preparation process of EBSD sample is first ground with metallographic sandpaper, and then it performs vibration polishing to ensure that there is no deformed layer on the surface of the sample.

The hardness of different areas of the sample is tested on the FM-800 Vickers microhardness tester (FUTURE–TECH, Kawasaki, Japan), with a load of 100 gf, three points are measured in each area, and the average value is taken.

## 3. Results

### 3.1. Creep Rupture of Welded Joints

**Figure 4:**
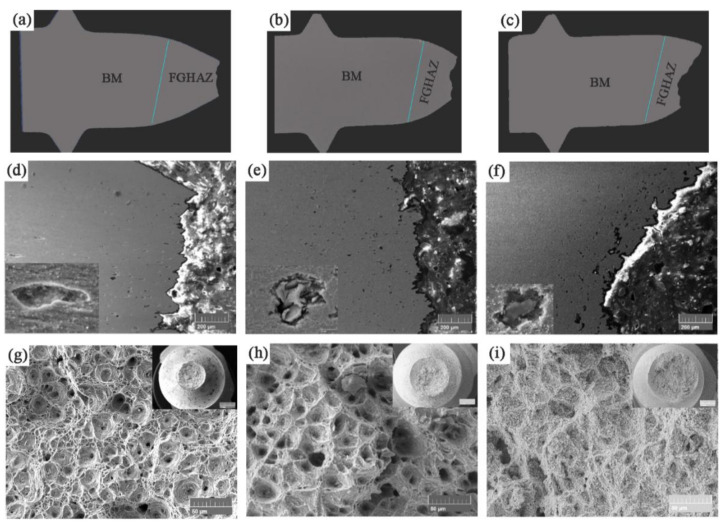
The microstructure of the fracture section and fracture morphology under different stresses: (**a**) 140 MPa macro section; (**b**) 120 MPa macro section; (**c**) 100 MPa macro section; (**d**) 140 MPa micro section; (**e**) 120 MPa micro section; (**f**) 100 MPa micro section; (**g**) 140 MPa fracture morphology; (**h**) 120 MPa fracture morphology; (**i**) 100 MPa fracture morphology.

In order to clarify the fracture location of the sample, the fractured creep sample was dissected to obtain the microstructure of the cross-section, as shown in Figure 4. The fracture locations of the samples are all located in FGHAZ as shown in Figure 4a–c. This is different from the typical fracture mode of 9Cr martensite and austenitic heat-resistant steel joints [19,20,21]. Studies have shown that the joint of 9Cr martensite and austenite fractures in the BM or weld under high stress, and fractures in the FGHAZ under low stress under creep [21]. This is related to the changes in the microstructure and performance degradation of FGHAZ during welding and post-weld heat treatment, such as hardness reduction and grain refinement. It also depends on external conditions such as temperature and applied stress [19]. The 9 h sample necked significantly, and there was a fibrous morphology formed by plastic flow in the area near the edge of the fracture, indicating that the fracture of the sample was controlled by plastic deformation. The fracture mode was similar to that of quasi-static tension. The large number of dimples (Figure 4g) on the fracture surface also indicates that the fracture process is accompanied by strong plastic deformation. In addition, some cavities (Figure 4d) can also be observed in the vicinity of the fracture. The edges of these cavities are relatively flat and deformed in the tensile direction. As the stress decreases, the creep rupture time increases, the fracture area shrinkage rate decreases (Figure 4h) and the number of cavities (Figure 4e) near the fracture location increases. The cavity changes from an ellipse along the stretching direction to a round shape, and the edge of the cavity becomes uneven. The dimple size increases and the depth decreases. When the creep stress is reduced to 100 MPa and the creep life is 719 h, the plastic deformation at the fracture location is very small (Figure 4c). The fracture location is still in FGHAZ, but the normal of the fracture plane is no longer parallel to the axis of the specimen, that is, it becomes an angle. The cavities near the fracture have a tendency to join together (Figure 4f). There are many white precipitates around the cavities. These precipitated phases hinder the grain boundary slip. At the same time, due to the high hardness and brittleness of the martensite microstructure, it is difficult for the martensite lath to slip during the creep deformation process, which causes the local stress near the precipitated phase to be large, and it is easy to form micro-cracks [22,23]. The dimple size becomes further shallower (Figure 4i). The size of these dimples is comparable to the size of prior-austenite grains. As the creep time increases, the fracture mode of the joint changes from toughness to brittleness. Under high stress (140 MPa), the creep rupture mechanism is overload rupture caused by the reduction of load-bearing section caused by dislocation slip. When the stress is small (100 MPa), the creep fracture mechanism is the accumulation of dislocations near the precipitated phases to form voids, the voids grow up and the connection fractures.

### 3.2. Microstructure Near the Fracture

**Figure 5:**
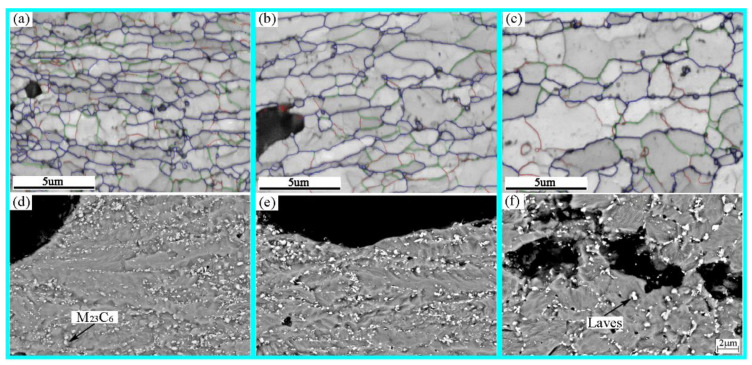
The microstructure near the fracture under different stress: (**a**) 140 MPa EBSD; (**b**) 120 MPa EBSD; (**c**) 100 MPa EBSD; (**d**) 140 MPa BSE; (**e**) 120 MPa BSE; (**f**) 100 MPa BSE.

The microstructure near the fracture was characterized by SEM and EBSD as shown in Figure 5. When the applied stress is 140 MPa, it can be seen from the EBSD IQ diagram (Figure 5a) that the shape of the prior-austenite grains elongates in the tensile direction. The grain boundaries can be vaguely seen from the SEM BSE image (Figure 5d). There are many gray carbides distributed on it, which is characterized as M_23_C_6_ by the energy spectrum. Its size is about 120–160 μm. Very fine Laves phase precipitated on it. When the applied stress is reduced to 120 MPa, the microstructure characteristics are similar to those at 140 MPa, but the size of the prior-austenite grains increases (Figure 5b). The precipitates and carbides are linearly distributed in chains, and the bright white Laves are obviously coarsened. Prior-austenite grains increase gradually (Figure 5b). When the applied stress is reduced to 100 MPa, the microstructure is fine tempered martensite. There are no carbides and precipitates distributed linearly in a chain. Most of the precipitated phases and carbides are distributed with the grain boundaries, and there are few intragranular. Many tiny cravities are formed around the Laves phase of the grain boundary. Prior-austenite grains evolve into equiaxed grains (Figure 5f).

**Figure 6:**
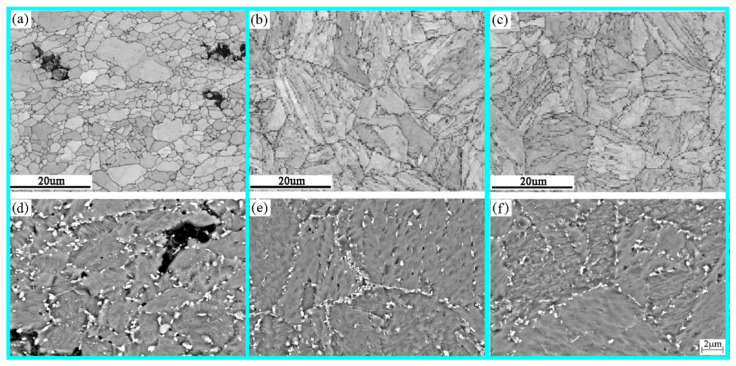
Microstructures at different positions under 100 MPa stress: (**a**) EBSD in near the fracture; (**b**) EBSD in BM; (**c**) EBSD in TE; (**d**) BSE in near the fracture; (**e**) BSE in BM; (**f**) BSE in TE.

Figure 6 shows the microstructure of the fracture, BM and TE under a stress of 100 MPa. The microstructure near the fracture is different from that of the BM. The microstructure near the fracture is fine and highly tempered martensite, which is also called decomposed lathless microstructure [24,25] (Figure 6a). These microstructures decompose due to the creep process. The grain size is about 5 μm. There are two large and small cavities distributed near the fracture (Figure 6d). The edge of the large cavity coincides with the grain boundary, and the size is equivalent to the grain size. The small cavities are attached to the bright white Laves phase, distributed along the grain boundaries, and tend to be connected together, with a size of about 240–400 nm. The BSE picture uses the atomic number as the contrast, while the atomic number of the Cr element and the Fe element is not much different. Therefore, the M_23_C_6_ in the picture is not so obvious that the gray display size is greater than 230–250 μm. In the BM, the prior-austenite grain boundary can be clearly seen, and the grain size is obviously larger than the fracture part, about 30 μm. The interface at this time is mainly the prior-austenite interface, the package boundary and the martensite lath boundary (Figure 6b). The bright white Laves phase and gray M_23_C_6_ carbides are densely distributed on the prior-austenite grain boundaries, and their sizes are significantly smaller than the precipitated phases near the fracture, which are about 200–270 μm and 160–200 μm (Figure 6e), respectively. At the boundary of the martensite lath bundle in the grain, there are also dense precipitation phases and carbides, mainly M_23_C_6_ carbides. At the TE, the microstructure characteristics are similar to those of the BM, and they are all martensite microstructures (Figure 6c). However, the size and quantity of precipitated phases and carbides are smaller, compared with the BM (Figure 6f). It shows that creep accelerates the nucleation and coarsening rate of the precipitated phase.

**Figure 7:**
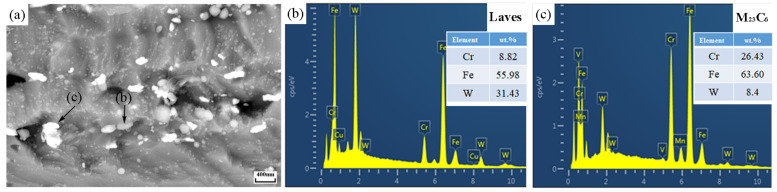
EDS characterization of Laves phase and M23C6: (**a**) Microstructure; (**b**) Laves; (**c**) M_23_C_6_.

The compositions of precipitates and carbides were characterized. It can be seen from Figure 7 that the bright white precipitated phase contains more W elements, which is the Laves phase; while the gray phase contains more Cr element, which is M_23_C_6_ carbide.

**Figure 8:**
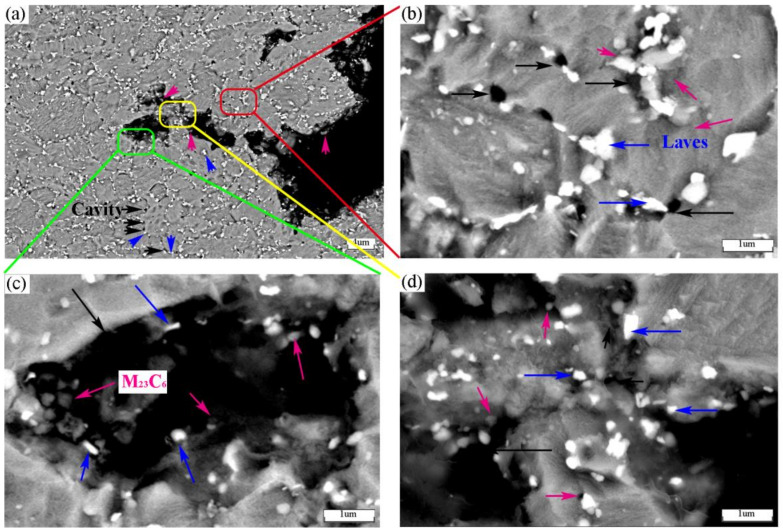
Precipitated phases near the 100 MPa fracture: (**a**) BSE pictures with lower magnification; (**b**) BSE pictures with smaller cavities; (**c**) BSE picture with bigger cavity; (**d**) Cavities in the trigeminal grain boundary.

The relationship between the cavity and the second phase was studied using the microstructure near the fracture under 100 MPa stress, as shown in Figure 8. First of all, it can be seen from Figure 8a that there are many cavities near the fracture. The large cavities coincide with the grain boundaries, while the small cavities are distributed in the grain boundaries. It can be seen from Figure 8b that these small cavities are attached to the Laves phase of the grain boundary. The coarse carbides appearing in the FGHAZ are related to the heat received during the welding process. Since the peak temperature experienced by this region during the welding process is slightly higher than AC3, this temperature is not enough to completely dissolve the carbides located at the grain boundaries of the prior-austenite grain boundary. In the subsequent tempering and creep process, these incompletely dissolved carbides will continue to grow as the core of carbide growth. Therefore, the carbide can grow to a larger size in a shorter time [26]. At the same time, these carbides will also nucleate separately, making the number of carbides increase. The coarsening of carbides not only reduces the precipitation strengthening effect on the matrix, but also causes strain concentration around the carbides due to the large difference in mechanical properties between the carbides and the surrounding matrix. When the strain concentration is large, the interface between the carbide and the surrounding matrix will fracture due to the uncoordinated deformation on both sides and form a cavity. It can be seen from Figure 8c that there are more fine M_23_C_6_ carbides distributed around the larger cavity, most of which are elliptical or circular. At the same time, a small number of Laves phases are also distributed. It can be seen from Figure 8d that each crystal grain is basically disconnected, but the relationship between the crack direction and the second phase is not very close at the trigeminal grain boundary. The number of Laves phases accumulated at this location is more than that of M_23_C_6_. Studies have shown [21] that the formation of cravity is related to the size of carbides. In the literature [27], the second phase particles are divided into three categories according to their diameters: large particles (*d* > 1 μm), medium particles (0.01 μm < *d* < 1 μm) and small particles (*d* < 1 μm). For the Laves phase in this paper, it is in the range of medium particles. Assuming that when the tensile stress at the interface between the Laves phase and the matrix reaches the critical value $\sigma_{c}$, the Laves phase separates from the matrix and a cavity is formed. It can be deduced that the macroscopic strain $\varepsilon_{c}$ used to characterize the initiation of cavity which is:

(2) $$\varepsilon_{C}=5.7\frac{d}{b}{\left[\frac{\sigma_{c}-\sigma_{o}-\sigma_{H}}{E}\right]}^{2}$$

where, *d* represents the particle diameter; *b* represents the modulus of the Burgers vector; *E* represents the elastic modulus of the matrix; *σ_o_* represents the yield strength; *σ_H_* represents the hydrostatic stress component. It can be seen from this formula that when the tensile stress reaches *σ_c_*, the larger the size of the Laves phase, the larger the macroscopic deformation of the initiating cavity, that is, the larger the cavity size, the more obvious the cavity. Cavities are easily formed around large particles under the same conditions. It is not easy to grow even after the cavity nucleate, since the sub-plastic zone is not easy to form around the small particles. Therefore, small particles are more likely to be broken down.

**Figure 9:**
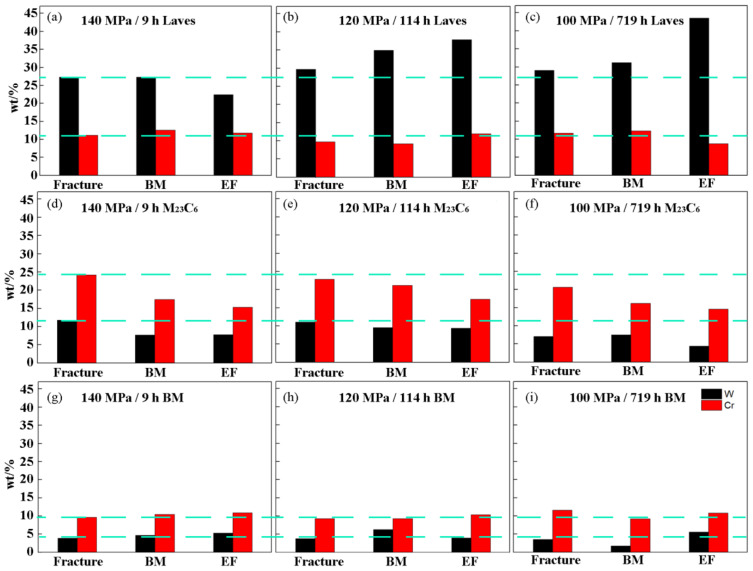
The statistical results of EDS W and Cr elements: (**a**) 140 MPa/9 h Laves; (**b**) 120 MPa/114 h Laves; (**c**) 100 MPa/719 h Laves; (**d**) 140 MPa/9 h M_23_C_6_; (**e**) 120 MPa/114 h M_23_C_6_; (**f**) 100 MPa/719 h M_23_C_6_; (**g**) 140 MPa/9 h BM; (**h**) 120 MPa/114 h BM; (**i**) 100 MPa/719 h BM.

Figure 9 shows the EDS statistical results of Laves phase, M_23_C_6_ phase and matrix in fracture, BM and TE under different creep times. In order to facilitate analysis, the Y-axis of each picture is set to the same value. The first line is the variation law of W and Cr content in Laves phase with different creep time and different positions. The second line is the change rule of W and Cr content in the M_23_C_6_ phase with different creep times and different positions. The third line is the change rule of W and Cr content in the matrix with different creep times and different positions. Firstly, the variation law of W contents in Laves phase with different creep time and different positions is analyzed. The W contents near the fracture increases slowly, while it increases greatly near the TE as the creep time increases, as shown in Figure 9a–c. The Cr content has small changes in different positions and different creep times. Then the source of W in the Laves phase of the creep process is analyzed. The W contents in the Laves phase increases with the increase of the creep time by comparing the three rows of pictures, regardless of the position of the fracture, the BM or the TE. In the M_23_C_6_ phase, the W contents decreases with the increase of creep time, and the W contents in the BM increases slowly with the increase of creep time. It shows that the W element in the Laves phase is mainly derived from M_23_C_6_. Finally, Cr contents in the M_23_C_6_ phase in the creep process is analyzed. The Cr contents near the fracture, the BM and the TE are all decreasing with the creep time increases as shown in Figure 9d–f. At the same time, the reduction of Cr contents near the fracture is smaller compared with the TE and BM.

**Figure 10:**
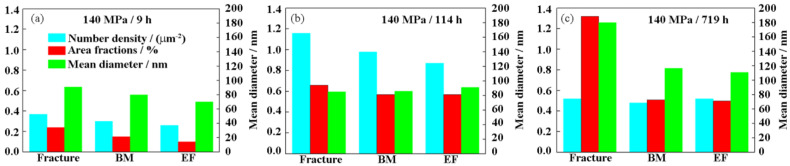
The size of Laves phase under different applied stresses: (**a**) 140 MPa; (**b**) 120 MPa; (**c**) 100 MPa.

The size of the Laves phase at different creep times and positions was quantitatively analyzed as shown in Figure 10. First of all, as a whole, the number density of the Laves phase at the fracture, BM and TE first increases and then decreases as the creep time increases. From creep 9 h to 114 h, the area fraction of Laves phase increases. From creep 114 h to 719 h, the area fraction of the fracture increased significantly, while the increase of BM and the TE was less. From creep 9 h to 114 h, the diameter of Laves phase increases slowly. From creep 114 h to 719 h, the diameter of the Laves phase increases significantly. The number density and area fraction near the fracture are higher than those of the BM and TE. The Laves phase is in the nucleation stage from creep 9 h to 114 h. It is in the coarsening stage from creep 114 h to 719 h. At the same time, the coarsening rate of the Laves phase near the fracture is greater than that of the BM and the TE. This is mainly related to the welding thermal cycle experienced in the welding process near the fracture. The Laves phase still does not reach thermodynamic equilibrium after creep for 100,000 h [10], while M_23_C_6_ can reach thermodynamic equilibrium after creep for 50,000 h and undergo Ostwald ripening with a constant volume fraction [28].

## 4. Discussion

### 4.1. The Nucleation Mechanism of Laves Phase

**Figure 11:**
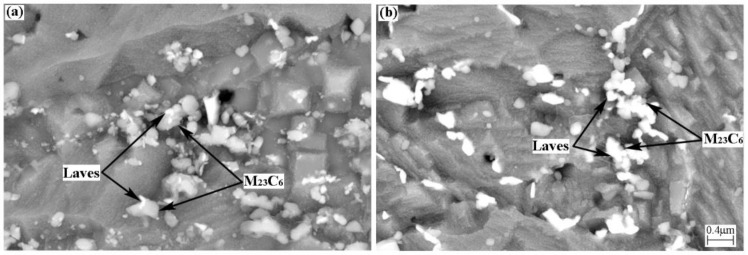
BSE image of BM with different creep times: (**a**) 9 h; (**b**) 114 h.

Figure 11 shows the BSE morphology of BM for different creep time. It can be seen from Figure 11a that the number of Laves phases is small and the diameter is also small, mainly nucleates on M_23_C_6_. The Laves phase basically occupies the entire M_23_C_6_ when the creep time is 114 h (Figure 11b). The same phenomenon can also be observed in FGHAZ. Different scholars have different opinions on the nucleation of Laves phase. We introduced two different views on Laves phase nucleation in the introduction. The current research results show that the Laves phase grows into M_23_C_6_. The findings of this study seem to support Xu et al.’s view that the Laves phase nucleates at the interface between M_23_C_6_ and the matrix and grows into M_23_C_6_. This may be due to the fact that M_23_C_6_ contains more W elements than the matrix.

### 4.2. The Influence of Laves Phase on Mechanical Properties

The precipitation strengthening effect of Laves phase depends on its distribution characteristics. The smaller the size, the greater the number density, and the stronger the strengthening effect. The formula describing the influence of precipitates on creep strength is similar to the Orowan stress formula [29], namely:

(3) $$\sigma_{orowan}=3.32Gb\frac{\sqrt{f_{p}}}{d_{p}}$$

where, *G* is the shear modulus, 64 GPa at 650 °C; *σ_Orowan_* is the Orowan stress; *b* is the Burgers vector mode, 0.25 nm; *f_p_* is the volume fraction of precipitates, and *d_p_* is the average particle diameter.

**Figure 12:**
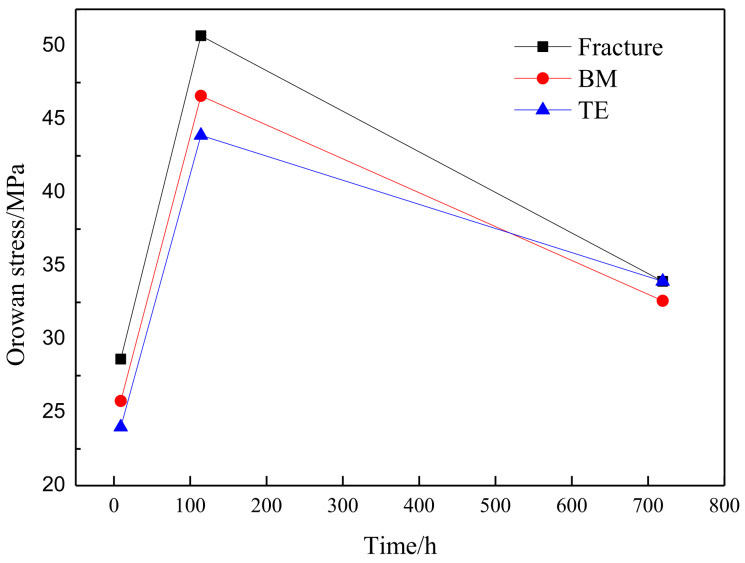
Orowan stress of Laves phase changes with creep time.

The Laves phase strengthening effect is estimated according to the above formula and the results are shown in Figure 12. The Orowan stress increased significantly from 9 h to 114 h. The Orowan stress near the fracture is the largest and the TE is the smallest due to the large area fraction of the Laves phase near the fracture. The Orowan stress begins to decrease from 114 h to 719 h. At this time, the Laves phase near the fracture is severely coarsened, resulting in a decrease in its strengthening effect. The strengthening effect decreased the most near the fracture, while the strengthening effect of the TE decreased the slowest.

The coarsening of the Laves phase at the grain boundary not only significantly reduces the precipitation strengthening effect but may become a cavity nucleation site when the grain boundary slides. From theoretical analysis, the larger the size of the grain boundary second phase particles, the more severe the stress-strain concentration at the interface with the surrounding matrix when creeping under external load, the easier it is to promote the formation of pores, resulting in a sudden drop in creep strength and brittle fracture along the grain [30,31].

**Figure 13:**
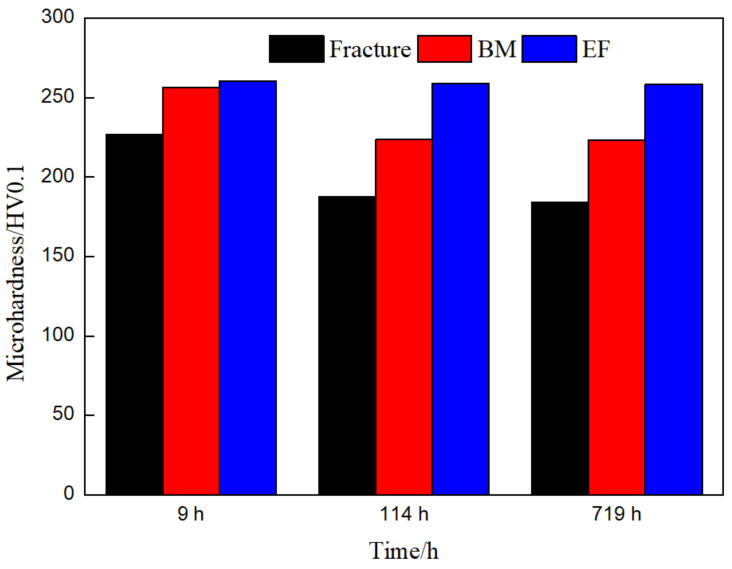
The microhardness of the joint in different parts.

Figure 13 shows the hardness changes of the fractured parts, the BM and the end face parts of the joint with different creep times. It can be seen that in the three creep times, the microhardness near the fracture is the lowest, being 227 HV, 188 HV and 183 HV, respectively; the end faces are the highest, with 261 HV, 259 HV and 258 HV, respectively; the hardness of the BM is between the end face and the fracture, respectively, 256 HV, 224 HV and 223 HV. The hardness near the fracture and the BM decreases first and then remains unchanged as the creep time increases. The hardness of the end face remained basically unchanged. It shows that aging has the least effect on the softening of the microstructure at the same temperature compared with creep. The softening of the microstructure is the most serious near the fracture due to the welding thermal process compared with the BM. The evolution of the microhardness is consistent with the results in the literature [6,32]. Some scholars [6,33] believe that the coarsening of precipitates is the reason why the microhardness decreases. In this study, it was found that the precipitates on the fracture, BM and the end face are all coarsening, and the coarsening rate of 114–719 h is obviously faster, but the decrease in hardness is not particularly obvious. Therefore, it is believed that the main reason of the decrease in hardness during creep is not the coarsening of precipitates. It is believed that the increase in sub-grain size is the main reason of the decrease in hardness, and the level of hardness does not depend on whether the sub-grain boundaries are pinned by carbides [6].

## 5. Conclusions

This paper mainly studies the microstructure and microhardness of G115/Sanicro25 dissimilar steel welded joints after creep at 675 °C. Our research provides the creep data of G115/Sanicr25 dissimilar steel welded joints, which is of great significance for understanding the role of Laves phase in heat-resistant steel welded joints. The conclusions obtained are as follows:

1. The fracture positions of the joints are all in the FGHAZ zone, and their microstructure is completely different from that of the base material. As the creep time increases, the fracture mode of the joint changes from toughness to brittleness. Under high stress (140 MPa), the creep rupture mechanism is overload rupture caused by the reduction of load-bearing section caused by dislocation slip. Under a stress of 100 MPa, the creep fracture mechanism is the accumulation of dislocations near the precipitated phases to form voids, the voids grow up and the connection fractures.
2. The Laves phase has obvious nucleation and coarsening stages. There are two ways to nucleate the Laves phase, the main way is to attach M_23_C_6_ to nucleate and to annex M_23_C_6_. Laves in the nucleation stage contribute to the creep strength, and the coarsening stage becomes worse. The longer the creep time, the worse the contribution of the Laves near the fracture to the creep strength.
3. There are two types of cavities in the fracture section. Small cavities sprout adjacent to the Laves phase; while large cavities occupy the entire sub-crystal, there are more precipitated phases around the cavities. As the size of the Laves phase increases, the cavities are more likely to nucleate.
4. Creep accelerates the coarsening rate of the Laves phase; aging increases the W content in the Laves phase.

## Figures and Tables

**Table 1:** Chemical composition of the WM and BMs (wt%).

Material	C	Cr	Co	W	V	Nb	B	Cu	Ni	Al	Ti	Mo	Fe
G115	0.094	8.84	3.01	2.7	0.17	0.06	0.013	0.84	0.03	0.008	0.01		Bal.
Sanicro25	0.08	22.5	1.5	3.6		0.5		3.0	25				Bal.
ErNiCrCoMo-1	0.062	22.43	11.18					0.01	54.72	1.22	0.405	8.82	Bal.

**Table 2:** G 115/Sanicro25 joint creep rupture test results.

Applied Stress (MPa)	Fracture Time (h)	Fracture Position
140	9	FGHAZ of G115
120	114	FGHAZ of G115
100	719	FGHAZ of G115

## Data Availability

No new data were created or analyzed in this study. Data sharing is not applicable to this article.
